# The absolute structure of ptilosarcenone 2.5-hydrate, a diterpenoid briarane from the orange sea pen *Ptilosarcus gurneyi* (Gray)

**DOI:** 10.1107/S1600536810050142

**Published:** 2010-12-18

**Authors:** Daniel J. Nurco, Douglas E. Conklin, Nathan S. Shapiro, Elaine Tran

**Affiliations:** a Department of Animal Science, University of California, One Shields Avenue, Davis, CA 95616, USA

## Abstract

In the title compound, C_24_H_29_ClO_8_·2.5H_2_O, which contains two organic mol­ecules (*A* and *B*) and five heavily disordered water mol­ecules in the asymmetric unit, the γ-lactone ring and the cyclo­hexenone ring are both *trans*-fused to the central cyclo­decene ring. The cyclehexenone ring features an α,β-unsaturated ketone with torsion angles between the conjugated carbonyl and alkene bonds of 0.6 (3) and 7.4 (4)° for mol­ecules *A* and *B*, respectively. The ptilosarcenone torsion angles between conjugated alkene bonds are 56.2 (5) and 55.4 (6)° for *A* and *B*, respectively. In the crystal, the components are linked by O—H⋯O hydrogen bonds. The absolute configuration of ptilosarcenone was determined unambiguously and exhibits similar absolute stereochemistry to that found in the crystal structures of other octocoralline briaranes.

## Related literature

In the 1970’s, two diterpenoid briaranes, ptilosarcone and ptilosarcenone, were purified from *Ptilosarcus gurneyi* (Wekell 1974 ▶; Wratten *et al.* 1977 ▶; Wekell 1978 ▶) and other octocorals have yielded similar compounds (Sung *et al.* 2002 ▶). In the presence of water or alcohol, ptilosarcone eliminates butyric acid, forming ptilosarcenone. Ptilosarcenone has also been found in extracts of *Tochuina tetraquetra*, a Tritoniid nudibranch that preys upon *Ptilosarcus gurneyi* (Williams & Andersen, 1987 ▶). For the structure of ptilosarcenone determined from a mostly complete room-temperature dataset, see: Hendrickson (1990 ▶); Hendrickson & Cardellina (1986 ▶).  Sea pens of the species *Ptilosarcus gurneyi* were collected near Juneau, Alaska (Smith, 2006 ▶) at depths of 5 to 10 m. For extraction and purification methods used, see: Wekell (1974 ▶). For related structures, see: Burks *et al.* (1977 ▶); Coval *et al.* (1988 ▶); Gonzalez *et al.* (2002 ▶); Grode *et al.* (1983 ▶); Hamann *et al.* (1996 ▶); van der Helm *et al.* (1986 ▶). For scientific background, see: Nurco (2008 ▶). 
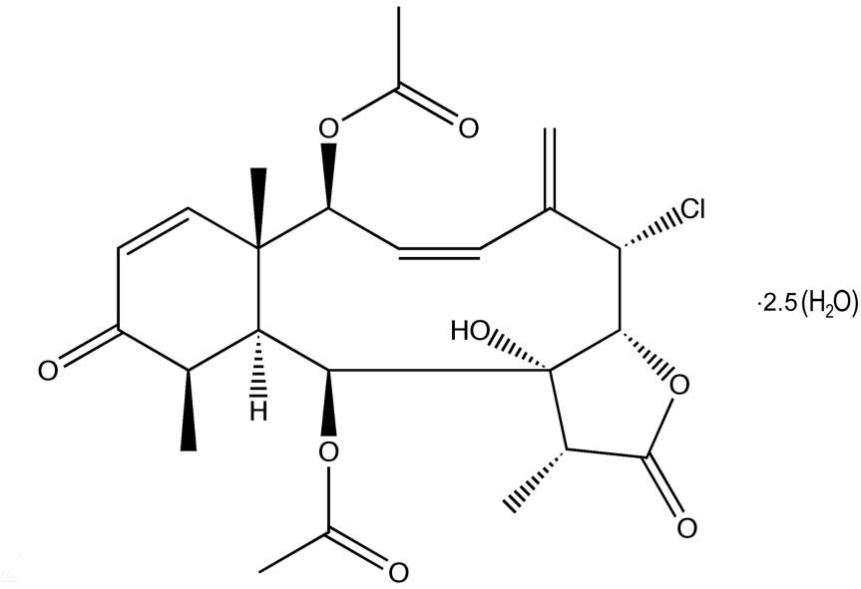


## Experimental

### 

#### Crystal data

2C_24_H_29_ClO_8_·5H_2_O
*M* *_r_* = 525.98
Orthorhombic, 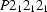

*a* = 9.8505 (4) Å
*b* = 13.5256 (6) Å
*c* = 39.3169 (17) Å
*V* = 5238.3 (4) Å^3^
*Z* = 8
Mo *K*α radiation
μ = 0.20 mm^−1^
*T* = 95 K
0.60 × 0.32 × 0.14 mm

#### Data collection

Bruker SMART APEXII diffractometer
Absorption correction: multi-scan (*SADABS*; Sheldrick, 1996 ▶) *T*_min_ = 0.888, *T*_max_ = 0.972
75112 measured reflections
13416 independent reflections
12944 reflections with *I* > 2σ(*I*)
*R*_int_ = 0.031

#### Refinement

*R*[*F* ^2^ > 2σ(*F* ^2^)] = 0.069
*wR*(*F* ^2^) = 0.172
*S* = 1.22
13416 reflections
673 parameters
12 restraints
H atoms treated by a mixture of independent and constrained refinement
Δρ_max_ = 0.92 e Å^−3^
Δρ_min_ = −0.60 e Å^−3^
Absolute structure: Flack (1983 ▶), 5967 Friedel pairs
Flack parameter: 0.07 (7)

### 

Data collection: *APEX2* (Bruker, 2007 ▶); cell refinement: *SAINT* (Bruker, 2007 ▶); data reduction: *SAINT*; program(s) used to solve structure: *SHELXS97* (Sheldrick, 2008 ▶); program(s) used to refine structure: *SHELXL97* (Sheldrick, 2008 ▶); molecular graphics: *SHELXTL* (Sheldrick, 2008 ▶); software used to prepare material for publication: *SHELXTL*.

## Supplementary Material

Crystal structure: contains datablocks I, global. DOI: 10.1107/S1600536810050142/hb5711sup1.cif

Structure factors: contains datablocks I. DOI: 10.1107/S1600536810050142/hb5711Isup2.hkl

Additional supplementary materials:  crystallographic information; 3D view; checkCIF report

## Figures and Tables

**Table 1:** Hydrogen-bond geometry (Å, °)

*D*—H⋯*A*	*D*—H	H⋯*A*	*D*⋯*A*	*D*—H⋯*A*
O6—H6*B*⋯O13	0.83 (2)	2.02 (3)	2.784 (4)	154 (6)
O14—H14*B*⋯O8^i^	0.85 (2)	2.13 (3)	2.932 (4)	156 (6)
O17—H17*A*⋯O3^ii^	0.81 (4)	2.04 (5)	2.834 (4)	167 (8)
O17—H17*B*⋯O16^iii^	0.80 (4)	2.31 (5)	3.082 (5)	161 (7)

Symmetry codes: (i) 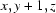; (ii) 
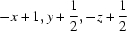
; (iii) 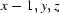.

**Fig. 1.**
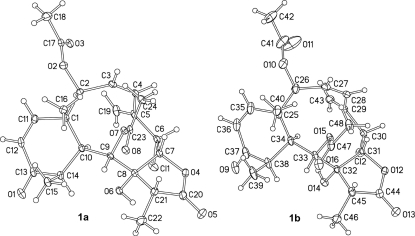
The molecular structure of both organic molecules in the asymmetric unit (1a and 1 b) drawn with 35% probability thermal ellipsoids.

## supplementary crystallographic information

### Comment

In subtidal waters along the west coast of North America lives the orange sea pen *Ptilosarcus gurneyi* (Gray). Sea pens are soft-bodied octocorals, cnidarians with 8-tentacled polyps, in the order pennatulacea. Like many acidacians, a single sea pen can be made up of thousands of specialized individuals each providing a specific function. They are also colonial organisms, measuring up to 1 meter in height and tending to live in large groups containing sometimes thousands of individual sea pens. The habit of a sea pen is to anchor into soft substrate of the ocean's bottom by its basal polyp. In the 1970's, two diterpenoid briaranes were purified from *Ptilosarcus gurneyi*, ptilosarcone and ptilosarcenone (Wekell 1974; Wratten *et al.* 1977; Wekell 1978). Other octocorals have yielded similar compounds (Sung *et al.* 2002). In the presence of water or alcohol ptilosarcone eliminates butyric acid, forming ptilosarcenone. Ptilosarcenone has also been found in extracts of *Tochuina tetraquetra*, a Tritoniid nudibranch that preys upon *Ptilosarcus gurneyi* (Williams & Andersen 1987). A crystal structure of ptilosarcenone from a room temperature dataset, mostly but not entirely complete, was reported in a dissertation (Hendrickson 1990) and referenced as unpublished data (Hendrickson & Cardellina 1986) but has not appeared in the peer-reviewed literature or the Cambridge Structural Database. Herein, we report a new crystallographic investigation of ptilosarcenone conducted with a low temperature dataset and featuring more favorable calculated results than the previous structure.

The structure (Figure 1) has two ptilosarcenone molecules and five disordered water molecules in the asymmetric unit. The γ-lactone and cyclohexenone rings are both *trans*-fused to the central cyclodecene ring. The cyclehexenone ring featured an α,β-unsaturated ketone with torsion angles between the conjugated carbonyl and alkene bonds of 0.6 (3)° and 7.4 (4)° for C11=C12—C13=O1 and C35=C36—C37=O9 in 1a and 1 b, respectively. The absolute configuration of ptilosarcenone was unambiguously determined with a Flack parameter of 0.06 (7) and revealed the following stereochemical assignments for 1a: C1(S), C2(S), *cis*-C3=C4, C6(S), C7(*R*), C8(*R*), C9(S), C10(S), *cis*-C11=C12, C14(*R*), C21(*R*), and 1 b: C25(S), C26(S), *cis*-C27=C28, C30(S), C31(*R*), C32(*R*), C33(S), C34(S), *cis*-C35=C36, C38(*R*), C45(*R*). The characterization of ptilosarcenone revealed similar absolute stereochemistry as found in structures of similar briaranes. Specifically, the absolute configuration at C6 (bearing a chlorine) and C8 (bearing a hydroxyl) was the same for ptilosarcenone as for briarein A (Burks, *et al.*, 1977), briantheins V (Coval, *et al.*, 1988), *X* (van der Helm *et al.*, 1986), and Y (Grode, *et al.*, 1983), 11-hydroxybrainthein (Gonzalez, *et al.*, 2002), and juncin E (Hamann, *et al.*, 1996). The ptilosarcenone C3=C4—C5=C19 and C27=C28—C29=C43 torsion angles between conjugated alkene bonds were 56.2 (5)° and 55.4 (6)° for 1a and 1 b. These values are similar to the analogous torsion angles found in the above mentioned compounds which were 69.0°, 57.0°, 57.3°, 48.7°, 48.5°, and 70.3° respectively, as ordered above.

### Experimental

Sea pens of the species *Ptilosarcus gurneyi* were collected near Juneau, Alaska (Smith, 2006) at depths of 5 to 10 meters using SCUBA techniques. Ptilosarcenone was purified from them *via* published procedures (Wekell, 1974). Crystals of ptilosarcenone were grown in a one half-dram vial from a binary solvent system using ethanol as the good solvent and water as the poor solvent.

### Refinement

Hydrogen atoms bonded to C atoms were generated by their idealized geometry and refined with a riding model, with C—H distances in the range 0.95 - 1.00 Å, and *U*_iso_ equal to 1.2*U*_eq_ or 1.5*U*_eq_ (methyl) of the bonded atom. Hydroxyl H6b and H14*b* were found on a difference map and refined with a restrained bond length of 0.84 (2) Å. H17*a* and H17*b*, bound to water molecule O17, were found in a difference map and refined with a restrained bond length of 0.84 (5) Å. The remaining water oxygen sites were severely disordered, and hydrogen atoms for these O atoms could not be located. For water oxygen atoms O18 through O28, isotropic thermal parameters were fixed at 0.036Å^2^, and their occupancies were restrained to sum to equal four water molecules.

### Crystal data

**Table:**

2C_24_H_29_ClO_8_·5H_2_O	*F*(000) = 2272
*M**_r_* = 525.98	*D*_x_ = 1.334 Mg m^−^^3^
Orthorhombic, *P*2_1_2_1_2_1_	Mo *K*α radiation, λ = 0.71073 Å
Hall symbol: P 2ac 2ab	Cell parameters from 9574 reflections
*a* = 9.8505 (4) Å	θ = 2.3–28.6°
*b* = 13.5256 (6) Å	µ = 0.20 mm^−^^1^
*c* = 39.3169 (17) Å	*T* = 95 K
*V* = 5238.3 (4) Å^3^	Block, colorless
*Z* = 8	0.60 × 0.32 × 0.14 mm

### Data collection

**Table:**

Bruker SMART APEXII diffractometer	13416 independent reflections
Radiation source: fine-focus sealed tube	12944 reflections with *I* > 2σ(*I*)
graphite	*R*_int_ = 0.031
Detector resolution: 8.3 pixels mm^-1^	θ_max_ = 28.6°, θ_min_ = 2.1°
ω scans	*h* = −13→13
Absorption correction: multi-scan (*SADABS*; Sheldrick, 1996)	*k* = −18→18
*T*_min_ = 0.888, *T*_max_ = 0.972	*l* = −52→52
75112 measured reflections	

### Refinement

**Table:**

Refinement on *F*^2^	Secondary atom site location: difference Fourier map
Least-squares matrix: full	Hydrogen site location: mixed
*R*[*F*^2^ > 2σ(*F*^2^)] = 0.069	H atoms treated by a mixture of independent and constrained refinement
*wR*(*F*^2^) = 0.172	*w* = 1/[σ^2^(*F*_o_^2^) + (0.0339*P*)^2^ + 10.7413*P*] where *P* = (*F*_o_^2^ + 2*F*_c_^2^)/3
*S* = 1.22	(Δ/σ)_max_ = 0.025
13416 reflections	Δρ_max_ = 0.92 e Å^−^^3^
673 parameters	Δρ_min_ = −0.60 e Å^−^^3^
12 restraints	Absolute structure: Flack (1983), 5967 Friedel pairs
Primary atom site location: structure-invariant direct methods	Flack parameter: 0.07 (7)

### Special details

**Table:**

Geometry. All e.s.d.'s (except the e.s.d. in the dihedral angle between two l.s. planes) are estimated using the full covariance matrix. The cell e.s.d.'s are taken into account individually in the estimation of e.s.d.'s in distances, angles and torsion angles; correlations between e.s.d.'s in cell parameters are only used when they are defined by crystal symmetry. An approximate (isotropic) treatment of cell e.s.d.'s is used for estimating e.s.d.'s involving l.s. planes.
Refinement. Refinement of *F*^2^ against ALL reflections. The weighted *R*-factor *wR* and goodness of fit *S* are based on *F*^2^, conventional *R*-factors *R* are based on *F*, with *F* set to zero for negative *F*^2^. The threshold expression of *F*^2^ > σ(*F*^2^) is used only for calculating *R*-factors(gt) *etc*. and is not relevant to the choice of reflections for refinement. *R*-factors based on *F*^2^ are statistically about twice as large as those based on *F*, and *R*-factors based on ALL data will be even larger.

### Fractional atomic coordinates and isotropic or equivalent isotropic displacement parameters (Å^2^)

**Table:**

	*x*	*y*	*z*	*U*_iso_*/*U*_eq_	Occ. (<1)
Cl1	0.84698 (9)	0.41736 (6)	0.22882 (2)	0.02499 (18)	
O1	0.2378 (2)	0.2518 (2)	0.23298 (7)	0.0259 (6)	
O2	0.6477 (3)	−0.01535 (19)	0.30844 (6)	0.0218 (5)	
O3	0.7263 (2)	0.0733 (2)	0.35230 (6)	0.0242 (5)	
O4	0.9534 (2)	0.28556 (19)	0.17337 (7)	0.0218 (5)	
O5	0.9390 (3)	0.3028 (2)	0.11727 (8)	0.0341 (7)	
O6	0.6462 (2)	0.28814 (18)	0.18938 (6)	0.0188 (5)	
H6B	0.681 (6)	0.328 (4)	0.1761 (12)	0.054 (18)*	
O7	0.7689 (2)	0.04304 (17)	0.20640 (6)	0.0154 (4)	
O8	0.7265 (2)	−0.05124 (18)	0.16024 (6)	0.0194 (5)	
C1	0.5560 (3)	0.0517 (2)	0.25706 (8)	0.0153 (6)	
C2	0.6687 (3)	0.0667 (2)	0.28492 (8)	0.0148 (6)	
H2	0.6514	0.1303	0.2971	0.018*	
C3	0.8169 (3)	0.0621 (2)	0.27476 (8)	0.0170 (6)	
H3	0.8563	−0.0019	0.2734	0.020*	
C4	0.8978 (3)	0.1380 (3)	0.26757 (8)	0.0190 (6)	
H4	0.9905	0.1230	0.2634	0.023*	
C5	0.8581 (3)	0.2438 (3)	0.26543 (9)	0.0196 (6)	
C6	0.9057 (3)	0.2917 (2)	0.23263 (9)	0.0188 (6)	
H6	1.0065	0.2969	0.2349	0.023*	
C7	0.8828 (3)	0.2328 (3)	0.20048 (9)	0.0178 (6)	
H7	0.9304	0.1682	0.2035	0.021*	
C8	0.7391 (3)	0.2092 (3)	0.18507 (8)	0.0174 (6)	
C9	0.6660 (3)	0.1143 (2)	0.19838 (8)	0.0153 (6)	
H9	0.6115	0.0869	0.1791	0.018*	
C10	0.5665 (3)	0.1325 (2)	0.22855 (8)	0.0148 (6)	
H10	0.5982	0.1943	0.2400	0.018*	
C11	0.4281 (3)	0.0707 (3)	0.27753 (9)	0.0181 (6)	
H11	0.4159	0.0309	0.2972	0.022*	
C12	0.3306 (3)	0.1369 (3)	0.27114 (9)	0.0214 (7)	
H12	0.2632	0.1489	0.2879	0.026*	
C13	0.3246 (3)	0.1909 (3)	0.23924 (9)	0.0189 (6)	
C14	0.4254 (3)	0.1575 (3)	0.21242 (9)	0.0167 (6)	
H14	0.4390	0.2137	0.1962	0.020*	
C15	0.3532 (4)	0.0736 (3)	0.19285 (9)	0.0224 (7)	
H15A	0.3306	0.0200	0.2086	0.034*	
H15B	0.4134	0.0484	0.1750	0.034*	
H15C	0.2698	0.0992	0.1825	0.034*	
C16	0.5534 (4)	−0.0566 (2)	0.24422 (9)	0.0182 (6)	
H16A	0.4945	−0.0962	0.2590	0.027*	
H16B	0.6456	−0.0838	0.2447	0.027*	
H16C	0.5186	−0.0583	0.2209	0.027*	
C17	0.6850 (3)	−0.0030 (3)	0.34076 (10)	0.0207 (7)	
C18	0.6728 (4)	−0.0981 (3)	0.36027 (11)	0.0319 (9)	
H18A	0.7601	−0.1328	0.3599	0.048*	
H18B	0.6031	−0.1398	0.3498	0.048*	
H18C	0.6473	−0.0837	0.3838	0.048*	
C19	0.7988 (4)	0.2904 (3)	0.29077 (10)	0.0257 (7)	
H19A	0.7800	0.2563	0.3114	0.031*	
H19B	0.7749	0.3581	0.2885	0.031*	
C20	0.8961 (4)	0.2672 (3)	0.14283 (10)	0.0248 (7)	
C21	0.7802 (4)	0.1953 (3)	0.14754 (9)	0.0213 (7)	
H21	0.8185	0.1272	0.1453	0.026*	
C22	0.6684 (4)	0.2056 (3)	0.12117 (10)	0.0320 (9)	
H22A	0.6250	0.2705	0.1235	0.048*	
H22B	0.6006	0.1535	0.1246	0.048*	
H22C	0.7073	0.1995	0.0983	0.048*	
C23	0.7878 (3)	−0.0371 (2)	0.18644 (8)	0.0155 (6)	
C24	0.8908 (4)	−0.1036 (3)	0.20204 (9)	0.0206 (7)	
H24A	0.8474	−0.1448	0.2194	0.031*	
H24B	0.9626	−0.0638	0.2126	0.031*	
H24C	0.9303	−0.1460	0.1844	0.031*	
Cl2	0.49246 (8)	0.78146 (7)	0.15901 (2)	0.02348 (17)	
O9	1.0339 (3)	1.0208 (2)	0.14272 (8)	0.0340 (7)	
O10	0.7227 (3)	0.9878 (2)	0.00946 (7)	0.0313 (6)	
O11	0.5472 (6)	1.0767 (4)	0.02727 (13)	0.090 (2)	
O12	0.5834 (2)	0.58739 (19)	0.12846 (6)	0.0208 (5)	
O13	0.6885 (3)	0.4573 (2)	0.15048 (7)	0.0269 (6)	
O14	0.7957 (2)	0.74182 (19)	0.14636 (6)	0.0197 (5)	
H14B	0.760 (6)	0.799 (2)	0.1455 (15)	0.053 (17)*	
O15	0.8084 (3)	0.6944 (2)	0.05473 (6)	0.0214 (5)	
O16	1.0020 (3)	0.6098 (2)	0.04541 (7)	0.0297 (6)	
C25	0.8689 (4)	0.9096 (3)	0.05107 (9)	0.0252 (8)	
C26	0.7180 (4)	0.9317 (3)	0.04118 (10)	0.0266 (8)	
H26	0.6763	0.9739	0.0593	0.032*	
C27	0.6255 (4)	0.8453 (3)	0.03428 (9)	0.0266 (8)	
H27	0.6335	0.8141	0.0127	0.032*	
C28	0.5334 (4)	0.8088 (3)	0.05569 (9)	0.0226 (7)	
H28	0.4756	0.7584	0.0471	0.027*	
C29	0.5119 (3)	0.8387 (3)	0.09151 (9)	0.0220 (7)	
C30	0.5067 (3)	0.7486 (3)	0.11485 (8)	0.0199 (6)	
H30	0.4201	0.7139	0.1091	0.024*	
C31	0.6193 (3)	0.6732 (3)	0.10824 (9)	0.0180 (6)	
H31	0.6116	0.6531	0.0838	0.022*	
C32	0.7731 (3)	0.6916 (3)	0.11541 (8)	0.0171 (6)	
C33	0.8587 (3)	0.7372 (3)	0.08619 (8)	0.0180 (6)	
H33	0.9532	0.7117	0.0892	0.022*	
C34	0.8698 (4)	0.8522 (3)	0.08562 (9)	0.0213 (7)	
H34	0.7894	0.8770	0.0986	0.026*	
C35	0.9248 (5)	1.0126 (3)	0.05641 (11)	0.0339 (9)	
H35	0.9248	1.0552	0.0372	0.041*	
C36	0.9743 (5)	1.0501 (3)	0.08541 (12)	0.0359 (10)	
H36	0.9922	1.1190	0.0868	0.043*	
C37	1.0015 (4)	0.9876 (3)	0.11488 (10)	0.0284 (8)	
C38	0.9963 (4)	0.8783 (3)	0.10774 (10)	0.0254 (7)	
H38	0.9862	0.8435	0.1300	0.030*	
C39	1.1368 (4)	0.8495 (4)	0.09277 (12)	0.0378 (11)	
H39A	1.1532	0.8872	0.0719	0.057*	
H39B	1.1377	0.7786	0.0876	0.057*	
H39C	1.2080	0.8644	0.1094	0.057*	
C40	0.9443 (4)	0.8610 (3)	0.02121 (10)	0.0287 (8)	
H40A	0.9730	0.9120	0.0050	0.043*	
H40B	0.8837	0.8141	0.0098	0.043*	
H40C	1.0243	0.8258	0.0298	0.043*	
C41	0.6288 (7)	1.0587 (3)	0.00541 (14)	0.0492 (13)	
C42	0.6379 (7)	1.1094 (4)	−0.02794 (15)	0.0553 (15)	
H42A	0.5550	1.0968	−0.0410	0.083*	
H42B	0.7166	1.0842	−0.0405	0.083*	
H42C	0.6482	1.1807	−0.0243	0.083*	
C43	0.4881 (4)	0.9301 (3)	0.10167 (10)	0.0259 (7)	
H43A	0.4837	0.9822	0.0855	0.031*	
H43B	0.4753	0.9438	0.1252	0.031*	
C44	0.6951 (4)	0.5343 (3)	0.13546 (9)	0.0210 (7)	
C45	0.8187 (3)	0.5830 (3)	0.12108 (9)	0.0228 (7)	
H45	0.8369	0.5530	0.0983	0.027*	
C46	0.9462 (4)	0.5708 (3)	0.14259 (12)	0.0341 (9)	
H46A	0.9335	0.6040	0.1645	0.051*	
H46B	1.0238	0.6002	0.1307	0.051*	
H46C	0.9634	0.5003	0.1464	0.051*	
C47	0.8881 (4)	0.6288 (3)	0.03764 (9)	0.0254 (8)	
C48	0.8111 (5)	0.5895 (4)	0.00796 (10)	0.0362 (10)	
H48A	0.7896	0.6437	−0.0077	0.054*	
H48B	0.7267	0.5588	0.0159	0.054*	
H48C	0.8663	0.5400	−0.0039	0.054*	
O17	0.2889 (4)	0.5904 (3)	0.07596 (8)	0.0351 (7)	
H17A	0.272 (8)	0.582 (6)	0.0959 (12)	0.08 (2)*	
H17B	0.224 (6)	0.592 (6)	0.0639 (16)	0.07 (2)*	
O18	0.5303 (4)	1.4924 (3)	0.05414 (11)	0.036*	0.753 (6)
O19	0.7874 (6)	1.4018 (4)	0.06457 (14)	0.036*	0.579 (7)
O20	0.4923 (8)	1.3333 (6)	0.00801 (19)	0.036*	0.419 (7)
O21	0.8447 (8)	1.2950 (6)	−0.03643 (19)	0.036*	0.413 (7)
O22	0.7887 (10)	1.3320 (7)	−0.0972 (3)	0.036*	0.327 (7)
O23	0.7397 (12)	1.2492 (9)	0.0247 (3)	0.036*	0.278 (7)
O24	0.7695 (11)	1.3529 (8)	−0.0514 (3)	0.036*	0.300 (6)
O25	0.5313 (13)	1.4108 (10)	0.0508 (3)	0.036*	0.247 (6)
O26	0.8040 (12)	1.3150 (8)	−0.0017 (3)	0.036*	0.287 (6)
O27	0.7929 (17)	1.3123 (12)	−0.0754 (4)	0.036*	0.196 (8)
O28	0.6751 (16)	1.3628 (12)	0.0420 (4)	0.036*	0.201 (7)

### Atomic displacement parameters (Å^2^)

**Table:**

	*U*^11^	*U*^22^	*U*^33^	*U*^12^	*U*^13^	*U*^23^
Cl1	0.0206 (4)	0.0162 (4)	0.0381 (5)	−0.0005 (3)	−0.0080 (4)	0.0002 (3)
O1	0.0143 (11)	0.0282 (13)	0.0353 (14)	0.0039 (10)	−0.0018 (10)	−0.0038 (11)
O2	0.0213 (12)	0.0237 (12)	0.0203 (12)	−0.0013 (10)	−0.0025 (10)	0.0051 (10)
O3	0.0137 (11)	0.0355 (15)	0.0234 (12)	−0.0005 (11)	−0.0016 (9)	0.0032 (11)
O4	0.0151 (11)	0.0228 (12)	0.0275 (12)	−0.0009 (10)	0.0036 (9)	0.0065 (10)
O5	0.0328 (15)	0.0389 (17)	0.0307 (15)	−0.0024 (13)	0.0093 (12)	0.0143 (13)
O6	0.0121 (10)	0.0169 (11)	0.0274 (12)	−0.0003 (9)	0.0004 (9)	0.0045 (10)
O7	0.0133 (10)	0.0147 (10)	0.0183 (11)	0.0017 (9)	−0.0009 (8)	−0.0014 (9)
O8	0.0162 (11)	0.0228 (12)	0.0193 (11)	0.0017 (9)	−0.0013 (9)	−0.0026 (10)
C1	0.0122 (13)	0.0159 (14)	0.0176 (14)	−0.0005 (12)	−0.0006 (11)	0.0008 (11)
C2	0.0136 (14)	0.0116 (13)	0.0194 (14)	0.0000 (11)	−0.0004 (11)	0.0040 (11)
C3	0.0160 (14)	0.0188 (15)	0.0161 (14)	0.0048 (12)	−0.0016 (11)	−0.0019 (12)
C4	0.0116 (13)	0.0286 (17)	0.0168 (15)	0.0004 (12)	−0.0016 (12)	0.0005 (13)
C5	0.0080 (13)	0.0225 (16)	0.0282 (17)	−0.0032 (12)	−0.0010 (12)	−0.0004 (13)
C6	0.0102 (13)	0.0164 (14)	0.0297 (17)	−0.0001 (12)	−0.0012 (12)	0.0030 (13)
C7	0.0117 (13)	0.0188 (15)	0.0231 (16)	0.0008 (12)	0.0010 (11)	0.0022 (13)
C8	0.0116 (13)	0.0209 (16)	0.0197 (15)	0.0001 (12)	−0.0013 (11)	0.0019 (12)
C9	0.0112 (13)	0.0187 (15)	0.0161 (14)	0.0036 (12)	−0.0010 (11)	0.0006 (11)
C10	0.0113 (13)	0.0131 (13)	0.0199 (14)	−0.0006 (11)	0.0001 (12)	−0.0002 (12)
C11	0.0161 (15)	0.0196 (15)	0.0187 (15)	−0.0045 (12)	0.0006 (12)	−0.0011 (12)
C12	0.0122 (14)	0.0263 (17)	0.0255 (17)	−0.0029 (12)	0.0043 (13)	−0.0035 (14)
C13	0.0114 (14)	0.0191 (15)	0.0262 (17)	−0.0028 (12)	0.0009 (12)	−0.0025 (13)
C14	0.0120 (14)	0.0179 (15)	0.0201 (15)	−0.0022 (12)	0.0002 (11)	−0.0007 (12)
C15	0.0149 (14)	0.0256 (17)	0.0269 (17)	−0.0032 (14)	−0.0017 (13)	−0.0029 (14)
C16	0.0202 (15)	0.0139 (14)	0.0205 (15)	−0.0021 (12)	0.0003 (12)	−0.0010 (12)
C17	0.0100 (13)	0.0271 (17)	0.0251 (16)	−0.0014 (12)	0.0010 (12)	0.0069 (14)
C18	0.034 (2)	0.030 (2)	0.032 (2)	−0.0027 (17)	−0.0037 (17)	0.0116 (16)
C19	0.0167 (15)	0.0274 (18)	0.0328 (19)	−0.0055 (14)	0.0026 (14)	−0.0050 (15)
C20	0.0178 (15)	0.0243 (18)	0.0323 (19)	0.0025 (14)	0.0048 (14)	0.0054 (15)
C21	0.0212 (16)	0.0212 (16)	0.0216 (16)	−0.0021 (13)	0.0038 (13)	0.0037 (13)
C22	0.034 (2)	0.040 (2)	0.0224 (18)	−0.0062 (18)	−0.0048 (16)	0.0053 (16)
C23	0.0090 (13)	0.0196 (15)	0.0181 (14)	0.0008 (11)	0.0032 (11)	−0.0018 (12)
C24	0.0181 (15)	0.0195 (16)	0.0241 (17)	0.0044 (13)	−0.0006 (13)	−0.0020 (13)
Cl2	0.0180 (3)	0.0302 (4)	0.0222 (4)	0.0001 (3)	0.0066 (3)	0.0009 (3)
O9	0.0271 (14)	0.0402 (17)	0.0348 (15)	−0.0139 (12)	0.0049 (12)	−0.0154 (13)
O10	0.0403 (16)	0.0263 (14)	0.0273 (14)	−0.0041 (12)	0.0031 (12)	0.0004 (11)
O11	0.129 (5)	0.065 (3)	0.077 (3)	0.057 (3)	0.050 (3)	0.027 (3)
O12	0.0182 (11)	0.0228 (12)	0.0213 (12)	−0.0035 (10)	0.0025 (9)	0.0025 (10)
O13	0.0289 (14)	0.0227 (13)	0.0292 (14)	0.0015 (11)	0.0055 (11)	0.0028 (11)
O14	0.0165 (11)	0.0253 (13)	0.0172 (11)	0.0004 (10)	0.0000 (9)	−0.0049 (10)
O15	0.0197 (11)	0.0287 (13)	0.0158 (11)	−0.0081 (10)	0.0021 (9)	−0.0069 (10)
O16	0.0278 (14)	0.0347 (15)	0.0266 (13)	−0.0044 (12)	0.0084 (11)	−0.0072 (11)
C25	0.0262 (18)	0.0267 (18)	0.0226 (17)	−0.0114 (15)	0.0054 (14)	−0.0057 (14)
C26	0.0297 (19)	0.0288 (19)	0.0214 (17)	−0.0101 (16)	0.0044 (14)	−0.0011 (14)
C27	0.0289 (19)	0.033 (2)	0.0180 (16)	−0.0015 (16)	−0.0049 (14)	0.0008 (14)
C28	0.0223 (17)	0.0213 (16)	0.0241 (16)	−0.0013 (13)	−0.0082 (13)	0.0027 (13)
C29	0.0121 (14)	0.0291 (18)	0.0247 (16)	−0.0049 (13)	−0.0032 (13)	0.0046 (14)
C30	0.0149 (14)	0.0269 (17)	0.0179 (14)	−0.0044 (13)	−0.0006 (12)	−0.0007 (12)
C31	0.0112 (14)	0.0248 (17)	0.0180 (15)	−0.0072 (12)	−0.0006 (11)	0.0023 (12)
C32	0.0136 (14)	0.0210 (15)	0.0167 (14)	−0.0042 (12)	0.0016 (12)	−0.0014 (12)
C33	0.0154 (14)	0.0213 (16)	0.0171 (14)	−0.0049 (12)	0.0021 (12)	−0.0059 (12)
C34	0.0163 (16)	0.0288 (18)	0.0188 (15)	−0.0095 (13)	0.0043 (12)	−0.0058 (13)
C35	0.038 (2)	0.034 (2)	0.030 (2)	−0.0180 (18)	0.0103 (17)	−0.0045 (17)
C36	0.037 (2)	0.031 (2)	0.039 (2)	−0.0168 (18)	0.0077 (18)	−0.0076 (18)
C37	0.0160 (15)	0.036 (2)	0.0332 (19)	−0.0090 (16)	0.0065 (15)	−0.0099 (16)
C38	0.0162 (15)	0.0327 (19)	0.0273 (17)	−0.0100 (15)	0.0042 (14)	−0.0088 (15)
C39	0.0167 (18)	0.052 (3)	0.045 (2)	−0.0121 (18)	0.0072 (17)	−0.019 (2)
C40	0.032 (2)	0.034 (2)	0.0204 (17)	−0.0089 (17)	0.0092 (15)	−0.0009 (15)
C41	0.076 (4)	0.021 (2)	0.050 (3)	0.007 (2)	0.014 (3)	0.0032 (19)
C42	0.085 (4)	0.024 (2)	0.057 (3)	0.007 (3)	0.008 (3)	0.011 (2)
C43	0.0199 (16)	0.0294 (19)	0.0284 (18)	0.0016 (15)	−0.0003 (14)	0.0043 (15)
C44	0.0195 (16)	0.0231 (17)	0.0204 (16)	−0.0001 (13)	0.0041 (13)	−0.0047 (13)
C45	0.0169 (16)	0.0284 (18)	0.0231 (17)	−0.0055 (14)	0.0038 (13)	−0.0018 (14)
C46	0.0224 (18)	0.031 (2)	0.049 (3)	0.0037 (16)	−0.0015 (17)	0.0114 (19)
C47	0.0285 (19)	0.0285 (19)	0.0192 (16)	−0.0109 (15)	0.0096 (14)	−0.0059 (14)
C48	0.035 (2)	0.048 (3)	0.0253 (19)	−0.019 (2)	0.0068 (16)	−0.0145 (18)
O17	0.0370 (17)	0.0396 (17)	0.0287 (16)	−0.0028 (14)	−0.0060 (13)	−0.0046 (14)

### Geometric parameters (Å, °)

**Table:**

Cl1—C6	1.801 (3)	O9—C37	1.225 (5)
O1—C13	1.213 (4)	O10—C41	1.341 (6)
O2—C17	1.333 (4)	O10—C26	1.461 (5)
O2—C2	1.459 (4)	O11—C41	1.202 (7)
O3—C17	1.197 (5)	O12—C44	1.342 (4)
O4—C20	1.350 (5)	O12—C31	1.451 (4)
O4—C7	1.459 (4)	O13—C44	1.199 (5)
O5—C20	1.192 (5)	O14—C32	1.411 (4)
O6—C8	1.416 (4)	O14—H14B	0.849 (19)
O6—H6B	0.83 (2)	O15—C47	1.362 (5)
O7—C23	1.351 (4)	O15—C33	1.453 (4)
O7—C9	1.434 (4)	O16—C47	1.191 (5)
O8—C23	1.209 (4)	C25—C35	1.512 (5)
C1—C11	1.517 (5)	C25—C40	1.537 (5)
C1—C16	1.550 (4)	C25—C34	1.565 (5)
C1—C10	1.569 (4)	C25—C26	1.566 (6)
C1—C2	1.572 (4)	C26—C27	1.506 (5)
C2—C3	1.515 (4)	C26—H26	1.0000
C2—H2	1.0000	C27—C28	1.333 (5)
C3—C4	1.331 (5)	C27—H27	0.9500
C3—H3	0.9500	C28—C29	1.480 (5)
C4—C5	1.486 (5)	C28—H28	0.9500
C4—H4	0.9500	C29—C43	1.321 (5)
C5—C19	1.315 (5)	C29—C30	1.526 (5)
C5—C6	1.517 (5)	C30—C31	1.528 (5)
C6—C7	1.511 (5)	C30—H30	1.0000
C6—H6	1.0000	C31—C32	1.561 (4)
C7—C8	1.572 (4)	C31—H31	1.0000
C7—H7	1.0000	C32—C33	1.553 (5)
C8—C21	1.542 (5)	C32—C45	1.553 (5)
C8—C9	1.562 (5)	C33—C34	1.560 (5)
C9—C10	1.558 (4)	C33—H33	1.0000
C9—H9	1.0000	C34—C38	1.560 (5)
C10—C14	1.565 (4)	C34—H34	1.0000
C10—H10	1.0000	C35—C36	1.340 (6)
C11—C12	1.337 (5)	C35—H35	0.9500
C11—H11	0.9500	C36—C37	1.459 (6)
C12—C13	1.453 (5)	C36—H36	0.9500
C12—H12	0.9500	C37—C38	1.505 (5)
C13—C14	1.518 (5)	C38—C39	1.553 (5)
C14—C15	1.544 (5)	C38—H38	1.0000
C14—H14	1.0000	C39—H39A	0.9800
C15—H15A	0.9800	C39—H39B	0.9800
C15—H15B	0.9800	C39—H39C	0.9800
C15—H15C	0.9800	C40—H40A	0.9800
C16—H16A	0.9800	C40—H40B	0.9800
C16—H16B	0.9800	C40—H40C	0.9800
C16—H16C	0.9800	C41—C42	1.483 (7)
C17—C18	1.503 (5)	C42—H42A	0.9800
C18—H18A	0.9800	C42—H42B	0.9800
C18—H18B	0.9800	C42—H42C	0.9800
C18—H18C	0.9800	C43—H43A	0.9500
C19—H19A	0.9500	C43—H43B	0.9500
C19—H19B	0.9500	C44—C45	1.495 (5)
C20—C21	1.511 (5)	C45—C46	1.523 (5)
C21—C22	1.519 (5)	C45—H45	1.0000
C21—H21	1.0000	C46—H46A	0.9800
C22—H22A	0.9800	C46—H46B	0.9800
C22—H22B	0.9800	C46—H46C	0.9800
C22—H22C	0.9800	C47—C48	1.490 (5)
C23—C24	1.489 (5)	C48—H48A	0.9800
C24—H24A	0.9800	C48—H48B	0.9800
C24—H24B	0.9800	C48—H48C	0.9800
C24—H24C	0.9800	O17—H17A	0.81 (4)
Cl2—C30	1.798 (3)	O17—H17B	0.80 (4)
			
C17—O2—C2	118.0 (3)	C44—O12—C31	109.9 (3)
C20—O4—C7	111.1 (3)	C32—O14—H14B	110 (4)
C8—O6—H6B	99 (4)	C47—O15—C33	118.9 (3)
C23—O7—C9	120.6 (3)	C35—C25—C40	108.9 (3)
C11—C1—C16	108.6 (3)	C35—C25—C34	109.5 (3)
C11—C1—C10	108.4 (3)	C40—C25—C34	116.6 (3)
C16—C1—C10	115.3 (3)	C35—C25—C26	101.8 (3)
C11—C1—C2	101.2 (3)	C40—C25—C26	110.5 (3)
C16—C1—C2	111.1 (3)	C34—C25—C26	108.4 (3)
C10—C1—C2	111.2 (3)	O10—C26—C27	105.6 (3)
O2—C2—C3	105.8 (2)	O10—C26—C25	106.3 (3)
O2—C2—C1	104.1 (2)	C27—C26—C25	118.1 (3)
C3—C2—C1	119.4 (3)	O10—C26—H26	108.8
O2—C2—H2	109.0	C27—C26—H26	108.8
C3—C2—H2	109.0	C25—C26—H26	108.8
C1—C2—H2	109.0	C28—C27—C26	125.8 (4)
C4—C3—C2	127.0 (3)	C28—C27—H27	117.1
C4—C3—H3	116.5	C26—C27—H27	117.1
C2—C3—H3	116.5	C27—C28—C29	126.7 (3)
C3—C4—C5	126.7 (3)	C27—C28—H28	116.7
C3—C4—H4	116.6	C29—C28—H28	116.7
C5—C4—H4	116.6	C43—C29—C28	124.7 (3)
C19—C5—C4	122.3 (3)	C43—C29—C30	124.0 (3)
C19—C5—C6	125.2 (3)	C28—C29—C30	111.0 (3)
C4—C5—C6	112.2 (3)	C29—C30—C31	113.9 (3)
C7—C6—C5	116.1 (3)	C29—C30—Cl2	112.7 (3)
C7—C6—Cl1	112.3 (2)	C31—C30—Cl2	112.7 (2)
C5—C6—Cl1	112.0 (2)	C29—C30—H30	105.5
C7—C6—H6	105.1	C31—C30—H30	105.5
C5—C6—H6	105.1	Cl2—C30—H30	105.5
Cl1—C6—H6	105.1	O12—C31—C30	105.3 (3)
O4—C7—C6	106.4 (3)	O12—C31—C32	105.3 (3)
O4—C7—C8	104.3 (3)	C30—C31—C32	124.5 (3)
C6—C7—C8	124.3 (3)	O12—C31—H31	106.9
O4—C7—H7	106.9	C30—C31—H31	106.9
C6—C7—H7	106.9	C32—C31—H31	106.9
C8—C7—H7	106.9	O14—C32—C33	111.2 (3)
O6—C8—C21	112.1 (3)	O14—C32—C45	106.6 (3)
O6—C8—C9	106.4 (2)	C33—C32—C45	108.9 (3)
C21—C8—C9	109.9 (3)	O14—C32—C31	112.7 (3)
O6—C8—C7	112.5 (3)	C33—C32—C31	117.2 (3)
C21—C8—C7	99.0 (3)	C45—C32—C31	99.0 (3)
C9—C8—C7	116.9 (3)	O15—C33—C32	106.7 (3)
O7—C9—C10	112.6 (3)	O15—C33—C34	114.1 (3)
O7—C9—C8	107.5 (2)	C32—C33—C34	116.4 (3)
C10—C9—C8	114.5 (3)	O15—C33—H33	106.3
O7—C9—H9	107.3	C32—C33—H33	106.3
C10—C9—H9	107.3	C34—C33—H33	106.3
C8—C9—H9	107.3	C33—C34—C38	105.9 (3)
C9—C10—C14	106.5 (3)	C33—C34—C25	120.5 (3)
C9—C10—C1	118.4 (3)	C38—C34—C25	112.1 (3)
C14—C10—C1	112.4 (2)	C33—C34—H34	105.8
C9—C10—H10	106.3	C38—C34—H34	105.8
C14—C10—H10	106.3	C25—C34—H34	105.8
C1—C10—H10	106.3	C36—C35—C25	126.8 (4)
C12—C11—C1	127.6 (3)	C36—C35—H35	116.6
C12—C11—H11	116.2	C25—C35—H35	116.6
C1—C11—H11	116.2	C35—C36—C37	121.6 (4)
C11—C12—C13	121.9 (3)	C35—C36—H36	119.2
C11—C12—H12	119.1	C37—C36—H36	119.2
C13—C12—H12	119.1	O9—C37—C36	123.0 (4)
O1—C13—C12	123.1 (3)	O9—C37—C38	122.4 (4)
O1—C13—C14	121.5 (3)	C36—C37—C38	114.5 (3)
C12—C13—C14	115.1 (3)	C37—C38—C39	106.7 (3)
C13—C14—C15	105.3 (3)	C37—C38—C34	110.7 (3)
C13—C14—C10	111.3 (3)	C39—C38—C34	116.3 (3)
C15—C14—C10	116.9 (3)	C37—C38—H38	107.6
C13—C14—H14	107.6	C39—C38—H38	107.6
C15—C14—H14	107.6	C34—C38—H38	107.6
C10—C14—H14	107.6	C38—C39—H39A	109.5
C14—C15—H15A	109.5	C38—C39—H39B	109.5
C14—C15—H15B	109.5	H39A—C39—H39B	109.5
H15A—C15—H15B	109.5	C38—C39—H39C	109.5
C14—C15—H15C	109.5	H39A—C39—H39C	109.5
H15A—C15—H15C	109.5	H39B—C39—H39C	109.5
H15B—C15—H15C	109.5	C25—C40—H40A	109.5
C1—C16—H16A	109.5	C25—C40—H40B	109.5
C1—C16—H16B	109.5	H40A—C40—H40B	109.5
H16A—C16—H16B	109.5	C25—C40—H40C	109.5
C1—C16—H16C	109.5	H40A—C40—H40C	109.5
H16A—C16—H16C	109.5	H40B—C40—H40C	109.5
H16B—C16—H16C	109.5	O11—C41—O10	121.4 (5)
O3—C17—O2	124.3 (3)	O11—C41—C42	125.4 (5)
O3—C17—C18	124.8 (4)	O10—C41—C42	113.2 (5)
O2—C17—C18	110.9 (3)	C41—C42—H42A	109.5
C17—C18—H18A	109.5	C41—C42—H42B	109.5
C17—C18—H18B	109.5	H42A—C42—H42B	109.5
H18A—C18—H18B	109.5	C41—C42—H42C	109.5
C17—C18—H18C	109.5	H42A—C42—H42C	109.5
H18A—C18—H18C	109.5	H42B—C42—H42C	109.5
H18B—C18—H18C	109.5	C29—C43—H43A	120.0
C5—C19—H19A	120.0	C29—C43—H43B	120.0
C5—C19—H19B	120.0	H43A—C43—H43B	120.0
H19A—C19—H19B	120.0	O13—C44—O12	121.4 (3)
O5—C20—O4	121.8 (4)	O13—C44—C45	127.8 (4)
O5—C20—C21	129.1 (4)	O12—C44—C45	110.8 (3)
O4—C20—C21	109.0 (3)	C44—C45—C46	114.4 (3)
C20—C21—C22	113.9 (3)	C44—C45—C32	103.6 (3)
C20—C21—C8	103.7 (3)	C46—C45—C32	114.9 (3)
C22—C21—C8	116.8 (3)	C44—C45—H45	107.8
C20—C21—H21	107.3	C46—C45—H45	107.8
C22—C21—H21	107.3	C32—C45—H45	107.8
C8—C21—H21	107.3	C45—C46—H46A	109.5
C21—C22—H22A	109.5	C45—C46—H46B	109.5
C21—C22—H22B	109.5	H46A—C46—H46B	109.5
H22A—C22—H22B	109.5	C45—C46—H46C	109.5
C21—C22—H22C	109.5	H46A—C46—H46C	109.5
H22A—C22—H22C	109.5	H46B—C46—H46C	109.5
H22B—C22—H22C	109.5	O16—C47—O15	123.9 (3)
O8—C23—O7	123.6 (3)	O16—C47—C48	127.1 (4)
O8—C23—C24	126.5 (3)	O15—C47—C48	109.0 (4)
O7—C23—C24	109.9 (3)	C47—C48—H48A	109.5
C23—C24—H24A	109.5	C47—C48—H48B	109.5
C23—C24—H24B	109.5	H48A—C48—H48B	109.5
H24A—C24—H24B	109.5	C47—C48—H48C	109.5
C23—C24—H24C	109.5	H48A—C48—H48C	109.5
H24A—C24—H24C	109.5	H48B—C48—H48C	109.5
H24B—C24—H24C	109.5	H17A—O17—H17B	114 (7)
C41—O10—C26	116.8 (4)		
			
C17—O2—C2—C3	80.7 (3)	C40—C25—C26—C27	65.5 (4)
C17—O2—C2—C1	−152.6 (3)	C34—C25—C26—C27	−63.5 (4)
C11—C1—C2—O2	67.0 (3)	O10—C26—C27—C28	−139.8 (4)
C16—C1—C2—O2	−48.2 (3)	C25—C26—C27—C28	101.5 (5)
C10—C1—C2—O2	−178.0 (2)	C26—C27—C28—C29	−6.3 (6)
C11—C1—C2—C3	−175.4 (3)	C27—C28—C29—C43	55.4 (6)
C16—C1—C2—C3	69.3 (4)	C27—C28—C29—C30	−130.3 (4)
C10—C1—C2—C3	−60.5 (4)	C43—C29—C30—C31	−139.7 (4)
O2—C2—C3—C4	−146.8 (3)	C28—C29—C30—C31	45.9 (4)
C1—C2—C3—C4	96.5 (4)	C43—C29—C30—Cl2	−9.7 (5)
C2—C3—C4—C5	−4.9 (6)	C28—C29—C30—Cl2	176.0 (2)
C3—C4—C5—C19	56.2 (5)	C44—O12—C31—C30	−155.7 (3)
C3—C4—C5—C6	−129.2 (4)	C44—O12—C31—C32	−22.5 (3)
C19—C5—C6—C7	−140.5 (3)	C29—C30—C31—O12	−170.5 (3)
C4—C5—C6—C7	45.1 (4)	Cl2—C30—C31—O12	59.4 (3)
C19—C5—C6—Cl1	−9.7 (4)	C29—C30—C31—C32	68.1 (4)
C4—C5—C6—Cl1	175.9 (2)	Cl2—C30—C31—C32	−62.0 (4)
C20—O4—C7—C6	−153.6 (3)	O12—C31—C32—O14	−80.2 (3)
C20—O4—C7—C8	−20.7 (4)	C30—C31—C32—O14	41.2 (4)
C5—C6—C7—O4	−171.8 (3)	O12—C31—C32—C33	148.9 (3)
Cl1—C6—C7—O4	57.6 (3)	C30—C31—C32—C33	−89.7 (4)
C5—C6—C7—C8	67.4 (4)	O12—C31—C32—C45	32.1 (3)
Cl1—C6—C7—C8	−63.3 (4)	C30—C31—C32—C45	153.5 (3)
O4—C7—C8—O6	−85.7 (3)	C47—O15—C33—C32	−109.6 (3)
C6—C7—C8—O6	36.1 (4)	C47—O15—C33—C34	120.4 (3)
O4—C7—C8—C21	32.9 (3)	O14—C32—C33—O15	−168.7 (3)
C6—C7—C8—C21	154.7 (3)	C45—C32—C33—O15	74.0 (3)
O4—C7—C8—C9	150.8 (3)	C31—C32—C33—O15	−37.1 (4)
C6—C7—C8—C9	−87.4 (4)	O14—C32—C33—C34	−40.1 (4)
C23—O7—C9—C10	125.4 (3)	C45—C32—C33—C34	−157.3 (3)
C23—O7—C9—C8	−107.6 (3)	C31—C32—C33—C34	91.5 (4)
O6—C8—C9—O7	−158.7 (2)	O15—C33—C34—C38	−145.7 (3)
C21—C8—C9—O7	79.7 (3)	C32—C33—C34—C38	89.3 (3)
C7—C8—C9—O7	−32.1 (4)	O15—C33—C34—C25	−17.4 (4)
O6—C8—C9—C10	−32.8 (3)	C32—C33—C34—C25	−142.3 (3)
C21—C8—C9—C10	−154.4 (3)	C35—C25—C34—C33	−162.0 (3)
C7—C8—C9—C10	93.8 (3)	C40—C25—C34—C33	−37.7 (5)
O7—C9—C10—C14	−148.3 (3)	C26—C25—C34—C33	87.7 (4)
C8—C9—C10—C14	88.6 (3)	C35—C25—C34—C38	−36.5 (4)
O7—C9—C10—C1	−20.5 (4)	C40—C25—C34—C38	87.8 (4)
C8—C9—C10—C1	−143.7 (3)	C26—C25—C34—C38	−146.8 (3)
C11—C1—C10—C9	−164.1 (3)	C40—C25—C35—C36	−125.7 (5)
C16—C1—C10—C9	−42.1 (4)	C34—C25—C35—C36	3.0 (6)
C2—C1—C10—C9	85.5 (3)	C26—C25—C35—C36	117.5 (5)
C11—C1—C10—C14	−39.2 (3)	C25—C35—C36—C37	10.7 (7)
C16—C1—C10—C14	82.8 (3)	C35—C36—C37—O9	−172.6 (4)
C2—C1—C10—C14	−149.6 (3)	C35—C36—C37—C38	11.6 (6)
C16—C1—C11—C12	−119.1 (4)	O9—C37—C38—C39	−93.2 (4)
C10—C1—C11—C12	6.8 (5)	C36—C37—C38—C39	82.7 (4)
C2—C1—C11—C12	123.9 (4)	O9—C37—C38—C34	139.4 (4)
C1—C11—C12—C13	10.3 (6)	C36—C37—C38—C34	−44.8 (4)
C11—C12—C13—O1	−179.4 (3)	C33—C34—C38—C37	−168.7 (3)
C11—C12—C13—C14	7.4 (5)	C25—C34—C38—C37	58.1 (4)
O1—C13—C14—C15	−85.5 (4)	C33—C34—C38—C39	69.3 (4)
C12—C13—C14—C15	87.9 (3)	C25—C34—C38—C39	−63.8 (5)
O1—C13—C14—C10	146.9 (3)	C26—O10—C41—O11	2.0 (8)
C12—C13—C14—C10	−39.8 (4)	C26—O10—C41—C42	−177.9 (4)
C9—C10—C14—C13	−172.0 (3)	C31—O12—C44—O13	−176.2 (3)
C1—C10—C14—C13	56.8 (3)	C31—O12—C44—C45	1.8 (4)
C9—C10—C14—C15	67.0 (3)	O13—C44—C45—C46	−36.8 (6)
C1—C10—C14—C15	−64.2 (4)	O12—C44—C45—C46	145.4 (3)
C2—O2—C17—O3	5.2 (5)	O13—C44—C45—C32	−162.6 (4)
C2—O2—C17—C18	−172.9 (3)	O12—C44—C45—C32	19.6 (4)
C7—O4—C20—O5	−179.6 (4)	O14—C32—C45—C44	86.7 (3)
C7—O4—C20—C21	−1.6 (4)	C33—C32—C45—C44	−153.2 (3)
O5—C20—C21—C22	−30.5 (6)	C31—C32—C45—C44	−30.3 (3)
O4—C20—C21—C22	151.8 (3)	O14—C32—C45—C46	−38.8 (4)
O5—C20—C21—C8	−158.5 (4)	C33—C32—C45—C46	81.2 (4)
O4—C20—C21—C8	23.7 (4)	C31—C32—C45—C46	−155.9 (3)
O6—C8—C21—C20	85.5 (3)	C33—O15—C47—O16	−6.2 (5)
C9—C8—C21—C20	−156.4 (3)	C33—O15—C47—C48	175.6 (3)
C7—C8—C21—C20	−33.4 (3)	O26—O21—O24—O27	−164.9 (12)
O6—C8—C21—C22	−40.8 (4)	O24—O21—O26—O23	117.6 (13)
C9—C8—C21—C22	77.3 (4)	O27—O21—O26—O23	101.3 (17)
C7—C8—C21—C22	−159.6 (3)	O28—O23—O26—O21	−154.2 (12)
C9—O7—C23—O8	4.1 (5)	O21—O24—O27—O22	−157 (2)
C9—O7—C23—C24	−174.6 (3)	O24—O21—O27—O22	96 (6)
C41—O10—C26—C27	89.6 (5)	O26—O21—O27—O22	120 (6)
C41—O10—C26—C25	−144.1 (4)	O26—O21—O27—O24	23.9 (18)
C35—C25—C26—O10	62.8 (4)	O18—O25—O28—O19	46.5 (16)
C40—C25—C26—O10	−52.8 (4)	O18—O25—O28—O23	−162.2 (14)
C34—C25—C26—O10	178.2 (3)	O26—O23—O28—O19	−85.1 (10)
C35—C25—C26—C27	−178.9 (3)	O26—O23—O28—O25	122.3 (17)

### Hydrogen-bond geometry (Å, °)

**Table:**

*D*—H···*A*	*D*—H	H···*A*	*D*···*A*	*D*—H···*A*
O6—H6B···O13	0.83 (2)	2.02 (3)	2.784 (4)	154 (6)
O14—H14B···O8^i^	0.85 (2)	2.13 (3)	2.932 (4)	156 (6)
O17—H17A···O3^ii^	0.81 (4)	2.04 (5)	2.834 (4)	167 (8)
O17—H17B···O16^iii^	0.80 (4)	2.31 (5)	3.082 (5)	161 (7)

Symmetry codes: (i) *x*, *y*+1, *z*; (ii) −*x*+1, *y*+1/2, −*z*+1/2; (iii) *x*−1, *y*, *z*.
